# Determination of blood glucose parameter from human blood serum by using a quartz crystal microbalance sensor coated with phthalocyanines compounds

**DOI:** 10.3906/kim-1911-69

**Published:** 2020-10-26

**Authors:** Evin ŞAHİN SADIK, Hamdi Melih SARAOĞLU, İlke GÜROL, Mehmet Ali EBEOĞLU, Fatma Emel KOÇAK

**Affiliations:** 1 Electrical and Electronics Engineering Department, Faculty of Engineering, Kütahya Dumlupınar University, Kütahya TURKEY; 2 Institute of the Marmara Research Center of the Scientific and Technological Research Council of Turkey, Kocaeli Turkey; 3 Department of Medical Biochemistry, Kütahya Health Sciences University, Kütahya TURKEY

**Keywords:** Phthalocyanines, blood glucose, human blood serum, liquid sensing, quartz crystal microbalance

## Abstract

Determining the blood glucose level is important for the prevention and treatment of diabetes mellitus. We developed a sensor system using Quartz Crystal Microbalance (QCM) to determine the blood glucose level from human blood serum. This study consists of two experimental stages: artificial glucose/pure water solution tests and human blood serum tests. In the first stage of the study, the QCM sensor with the highest performance was identified using artificial glucose solution concentrations. In the second stage of the study, human blood serum measurements were performed using QCM to determine blood glucose levels. QCM sensors were coated with phthalocyanines (Pcs) by jet spray method. The blood glucose values of 96 volunteers, which ranged from 71 mg/dL to 329 mg/dL, were recorded. As a result of the study, human glucose values were determined with an average error of 3.25%.

## 1. Introduction

Diabetes is a noncontagious chronic disease and one of the largest global health problems in the 21st century. Researchers have shown that there were 415 million adults with diabetes around the world in 2015 and it is estimated that there will be 642 million adults with diabetes by 2040 [1]. The development of a low-cost glucose sensing device with high sensitivity, fast response time, good selectivity, and long-term stability will offer an enormous benefit for a significant portion of the human population in future generations. Besides, reliable measurement and continuous monitoring of blood glucose levels are essential for the diagnosis and treatment of diabetes. In line with this purpose, sensor applications are considered crucial for measurements of blood glucose levels.

The need for developing user-friendly, reliable, and compact sensor methods with high sensitivity, selectivity, and simultaneous observation ability is increasing day by day in analytical and physical chemistry, medical diagnosis, and biotechnology fields. Quartz crystal microbalance (QCM) becomes prominent among other sensor applications with some advantages like low-cost, rapid response times, and high sensitivity in gaseous and liquid-based studies.

The use of piezoelectric quartz crystals in sensor applications dates back to 1960 [2]. It has been shown in studies that the vibrational frequency of the crystal sensor changes the viscosity, density, and other characteristics of liquids [3–6]. Over the years, sensor applications on QCM have gained momentum in biomedical and biochemical fields and various sensor materials and sensor coatings have been utilized to detect various chemicals in liquid and gaseous media [7–12].

Phthalocyanines (Pcs) are well-established sensing materials for sensors such as QCM, surface acoustic wave (SAW), or conductive sensors due to their superb sensing qualities and wide diversity in their sorption properties. Furthermore, Pcs are very stable organic macromolecules and are employed in sensors among others due to their interesting chemical and physical properties [13]. Due to this fact, Pcs are used as a coating material. In the past decade, a new generation of Pcs-containing electron-withdrawing substituents such as fluorine has been developed [14]. Those Pcs are of much interest due to their high solubility in polar organic solvents. The electronic properties are also widely affected by introducing electron donor or acceptor groups. From this perspective, we evaluated four different phthalocyanines (Pcs) that are thought to be able to detect glucose. Also, there have been several studies in recent years on Pcs where they are used as a sensing material or in a novel glucose sensing application. Panda et al. suggested a graphene-coated SPR (surface plasmon resonance) sensor system that offered sensing of both glucose concentrations in human blood samples in the range 25–175 mg/dL and gas with refractive index variations from 1.0000 to 1.0007 at a wavelength of 589 nm. In this study, it has been perceived that a higher resonance angle shift is obtained for higher glucose concentrations [15]. Juan et al. used three microwave sensors to track the glucose levels of diﬀerent human blood plasma solutions. In this work, it has been observed that when trying to purify plasma, the sensitivity for complex solutions such as blood plasma is reduced [16]. Al-Sagur et al. fabricated an amperometric biosensor with immobilization of glucose oxidase onto the synthesized conducting hydrogel. The fabricated amperometric biosensor resulted in remarkable sensitivity and showed wide linear glucose detection [17].

The rest of the paper is organized as follows: QCM sensors are discussed in section II; the preparation of QCM sensors, coating chemicals, and measurement setup are introduced in section III. Section IV provides the experimental results. Finally, concluding remarks are given in section V.

## 2. Quartz crystal microbalance

**Figure 1:**
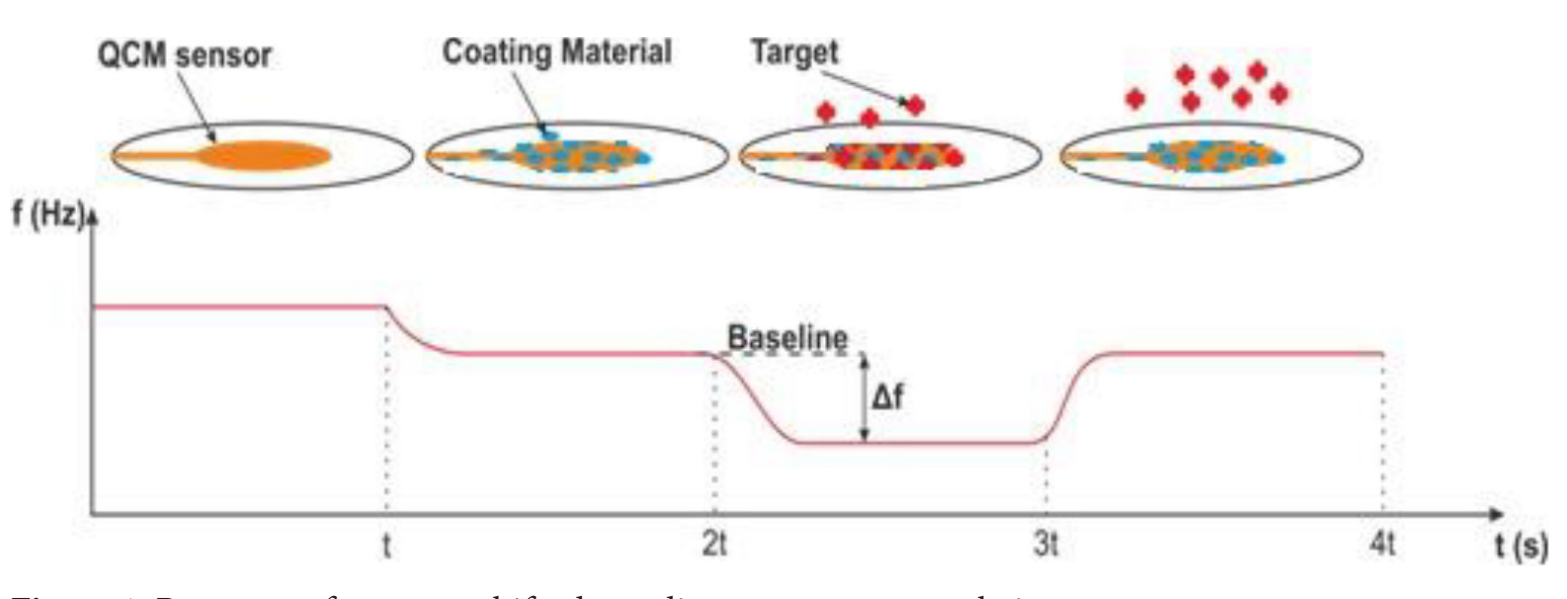
Resonance frequency shifts depending on mass accumulation.

All sensors can be considered to have two system components including a binding/recognition element and a signal transducer element [18]. QCM, used in sensor applications, has piezoelectric properties and is derived from a thin AT-cut quartz crystal. When electrical power is applied to the electrodes on the crystal, mechanical power is obtained due to the piezoelectric effect, and quartz crystal resonates at its natural frequency with the help of a crystal oscillator. The resonance frequency varies depending on the mass accumulation on the sensor surface (Figure 1). The washing solution (pure water) is applied to determine the initial resonance frequency value (baseline) of the measurement. When the target liquid is applied to the sensor, the resonance frequency changes in direct proportion to the accumulated mass. Thus, this frequency shift (Δf) gives the sensor response. The relation between the frequency and mass on the sensor is expressed by the Sauerbrey equation (1) [19]. According to Figure 1, an uncoated sensor shows a frequency value. As can be seen from Figure 1, the uncoated sensor produces a natural frequency. The figure shows that the sensor produces its natural frequency in the 0–t range. The frequency value of the covered sensor in the time interval of t–2t is seen. A frequency shift (∆f) proportional to the mass of the target analysis clinging to the coated sensor is observed in the 2t–3t time interval. Finally, it is seen that the frequency of the sensor returns to the baseline by movingthe analyte away from the sensor surface in the 3t–4t range.

(1)Δf=-202fAρqμqΔm

In the equation, f0 is the fundamental resonance frequency, Δf is the frequency variation, Δm is the mass change on the sensor surface, A is the area of the crystal surface, ρ_q_ is the quartz density, and μ_q_ is the cut off coefficient. As can be seen from the Sauerbrey equation, there is a theoretically linear relationship between the mass change on the sensor surface and the change in resonance frequency [20,21].

## 3. Experimental details 

### 3.1. Preparation of sensors

**Figure 2:**
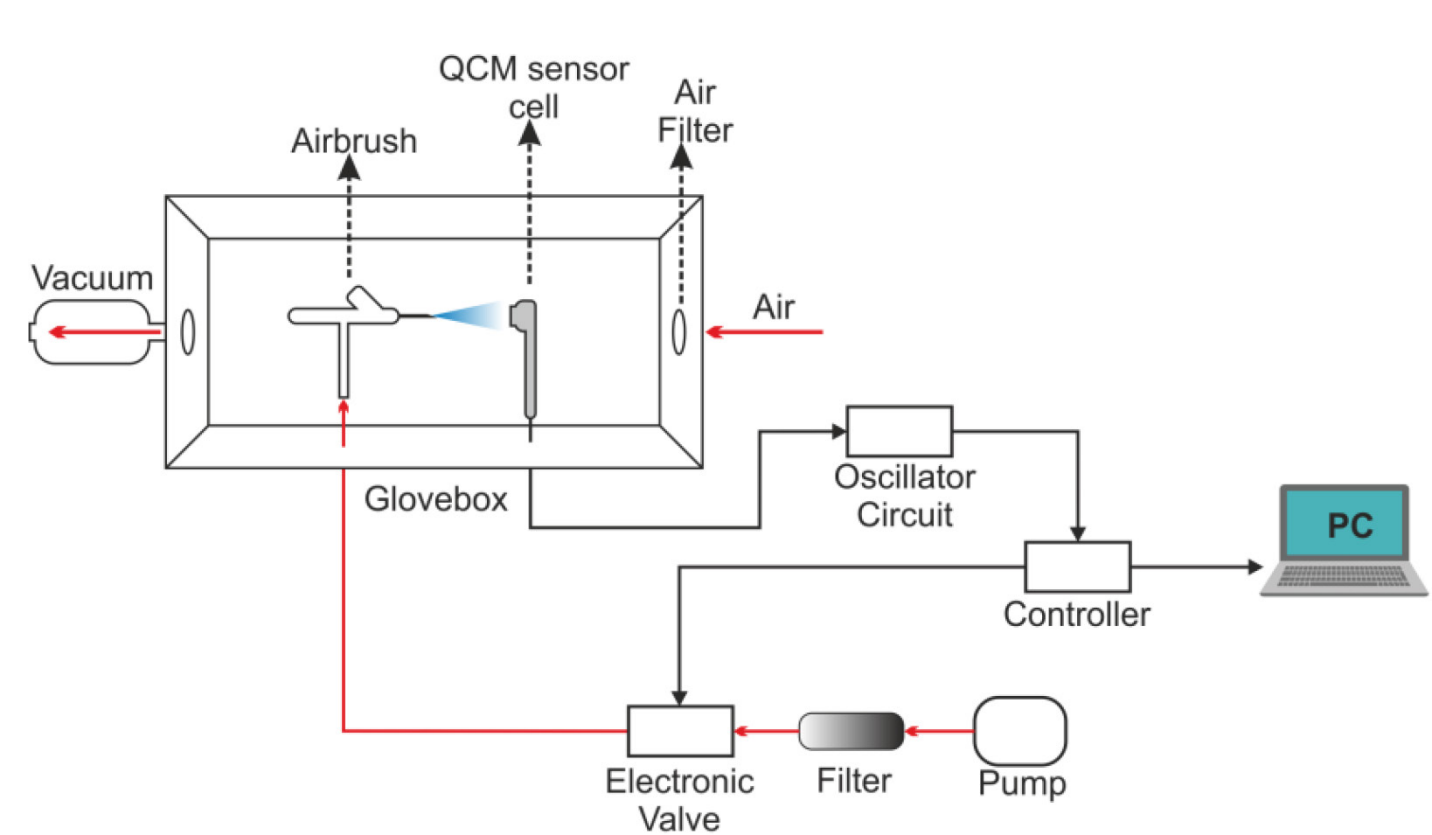
Schematic representation of the coating system.

In this study, Quartz crystals with AT-cut, gold electrode, diameter of 2.54 cm, and frequency of 5 MHz were used. The contact points of the electrodes of the sensor are located under the crystal for ease-of-use. The jet spray method is used as the QCM sensor coating method and the coating system is shown in Figure 2. The sensors are first cleaned with pure water and dried with dry air to prepare for the coating process. Phthalocyanines were obtained as coating materials from the Sensor Institute of the Marmara Research Center of the Scientific and Technological Research Council of Turkey (TÜBİTAK). Phthalocyanine dissolved in acetone at a rate of 1 mg/mL. Solvents were stuck to the sensor surface and then kiln-dried at 50 °C for 17 h to evaporate acetone residues.

**Figure 3:**
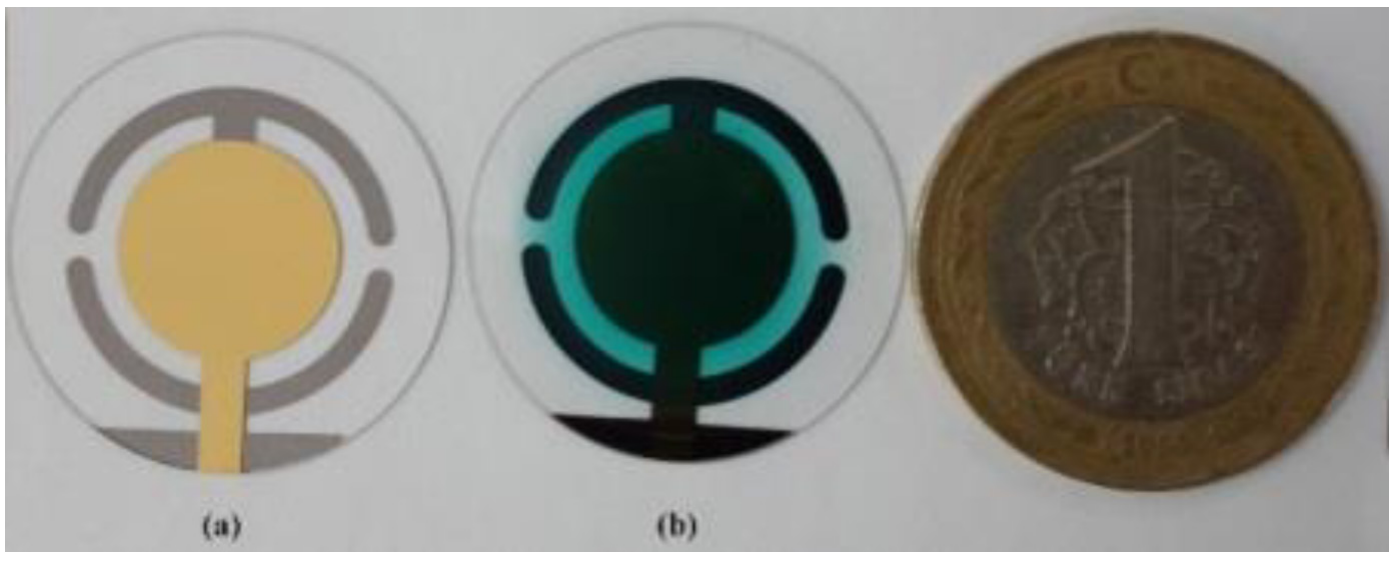
(a) Uncoated QCM sensor. (b) Coated QCM sensor.

The uncoated and coated QCM sensors are shown in Figure 3. It can be seen that a homogenous coating layer covered the gold electrode of the QCM sensor. QCM frequency was monitored simultaneously during the coating process, the frequency shift was observed, and the coating system was stopped when the desired frequency shift was obtained. The amount of coating material to shift the QCM frequency to 5 kHz was determined [22].

**Figure 4:**
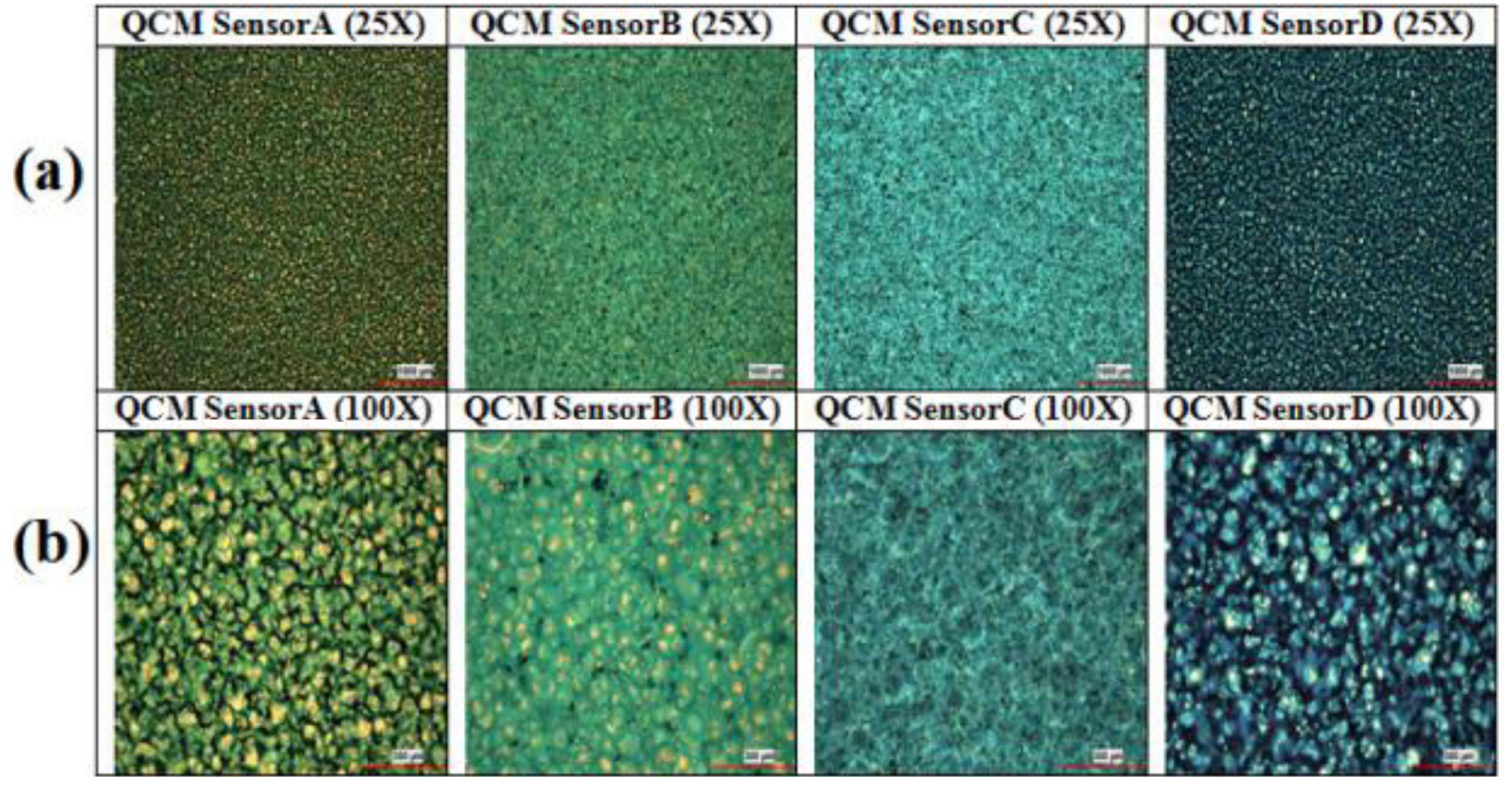
Surface photographs of the coated sensors under the electron microscope.

Four different QCM sensors were initially coated with four different phthalocyanine compounds for this study. QCM sensors were coded as Sensor A, Sensor B, Sensor C, and Sensor D. Surface photographs of the four coated QCM sensors were taken at 25× and 100× zoom by an electron microscope, which are shown in Figure 4. When Figure 4 is examined, it can be seen that the coating materials are distributed homogeneously on the sensors. One can say that with the coating system given in Figure 2, the homogeneity of the coating material on the sensor surfaces was achieved successfully. 

### 3.2. Chemicals 

**Figure 5:**
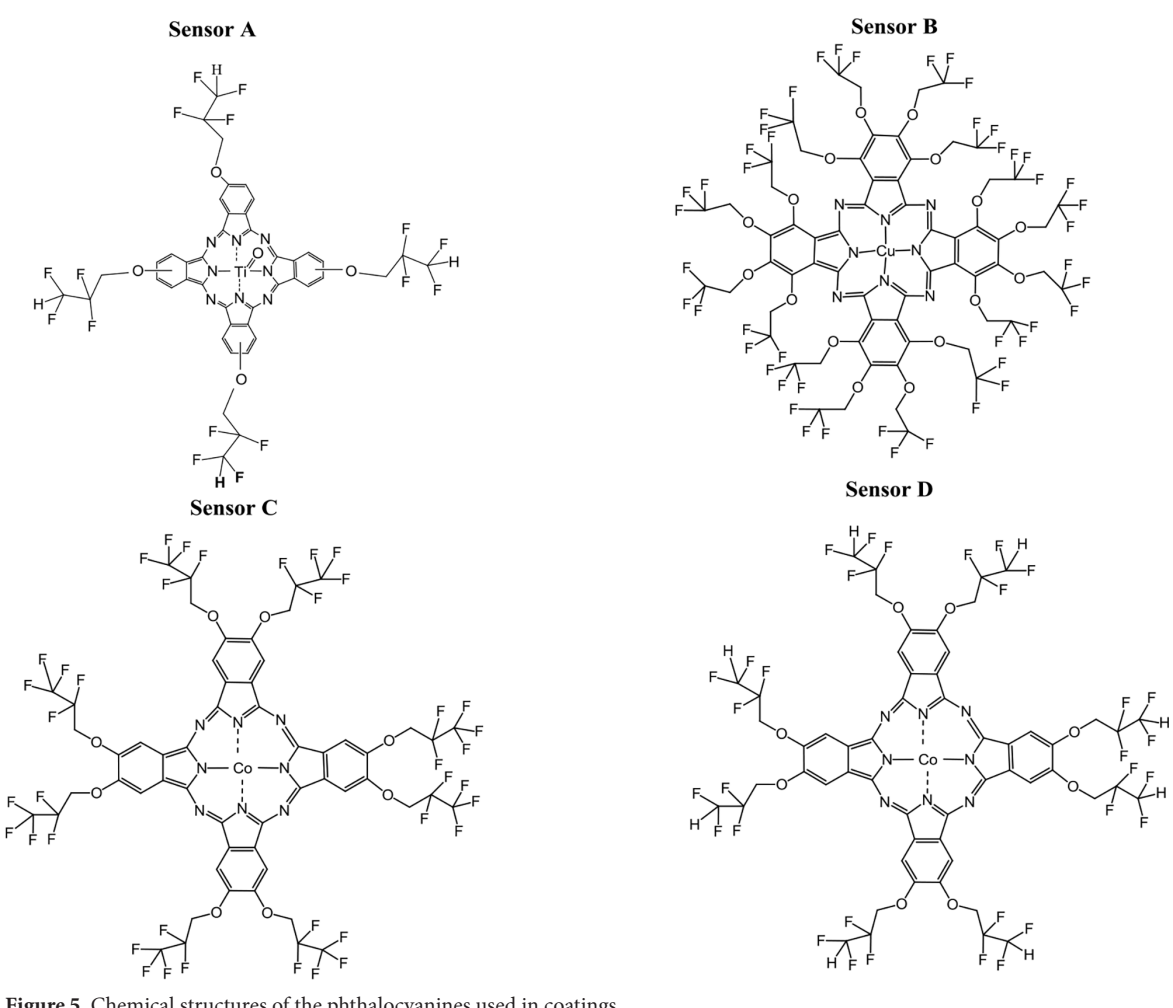
Chemical structures of the phthalocyanines used in coatings.

In this study, four different phthalocyanines were used as coating materials. Figure 5 shows the chemical structures of the coating materials for Sensor A, Sensor B, Sensor C, and Sensor D, respectively. 

### 3.3. Materials and instruments

**Figure 6:**
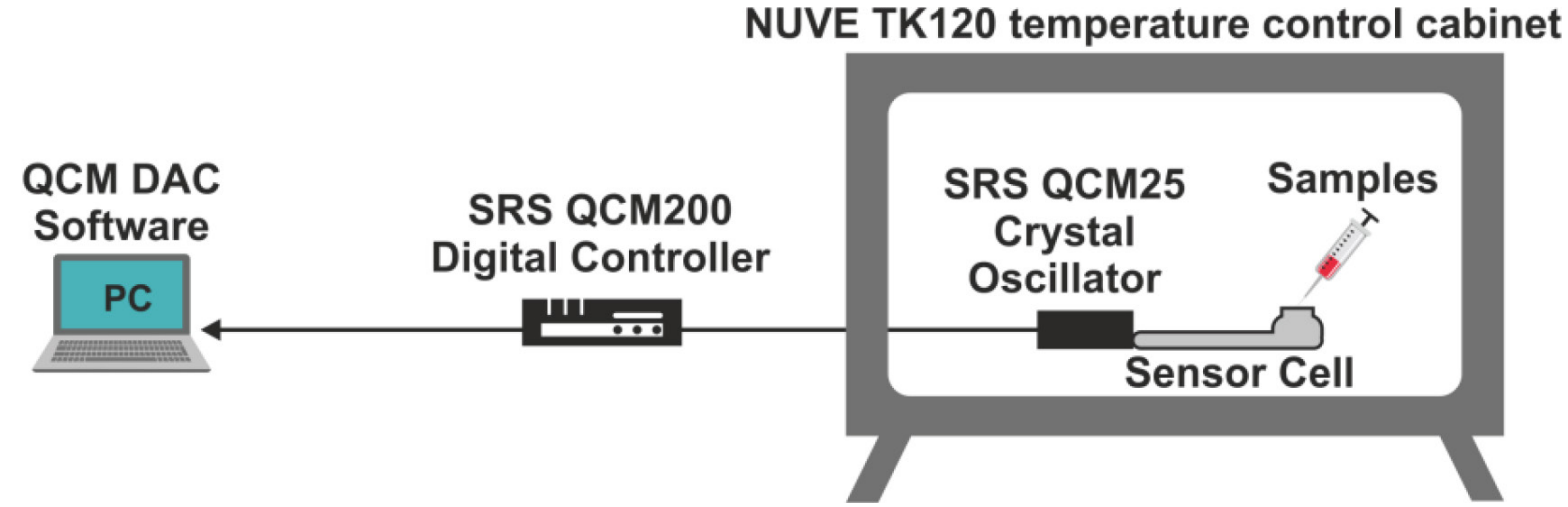
Schematic representation of the measurement setup.

Artificial glucose/pure water solutions were used with four different concentrations of 1.0 mg/dL, 2.5 mg/dL, 5.0 mg/dL, and 7.5 mg/dL for the experiments. In the main stage of the experiments, 96 human blood serums in the range of 71–329 mg/dL were used. The blood serum samples were taken from Dumlupınar University Evliya Çelebi Training and Research Hospital. The ethics committee approval was obtained from the Ethics Committee of the Medical Faculty at Uludağ University. The experimental setup used for the measurements is shown in Figure 6. The QCM sensor signals were recorded with the QCM200 measurement system based on frequency (SRS –Stanford Research Systems Inc., USA). All measurements were realized at a constant temperature of 4 °C. A temperature-controlled cabinet was used for the constant temperature (NUVE TK). The device measures the frequency and resistance of the 5 MHz AT cut crystal placed in the crystal cell. The resonance frequency varies according to the mass that accumulates on the crystal surface. The samples injected with the aid of a micropipette into the crystal cell changed the resonance frequency of the crystal and this change was recorded simultaneously. In the experiment, glucose/pure water solutions at different concentrations were used first, and after the glucose-sensitive sensor was identified, blood serum samples with different glucose values were applied to the QCM sensor in the crystal cell. After each measurement, pure water was also applied to sensors’ surfaces for cleaning purposes.

## 4. Results and discussion

### 4.1. Experiments with artificial glucose/pure water solutions

**Figure 7:**
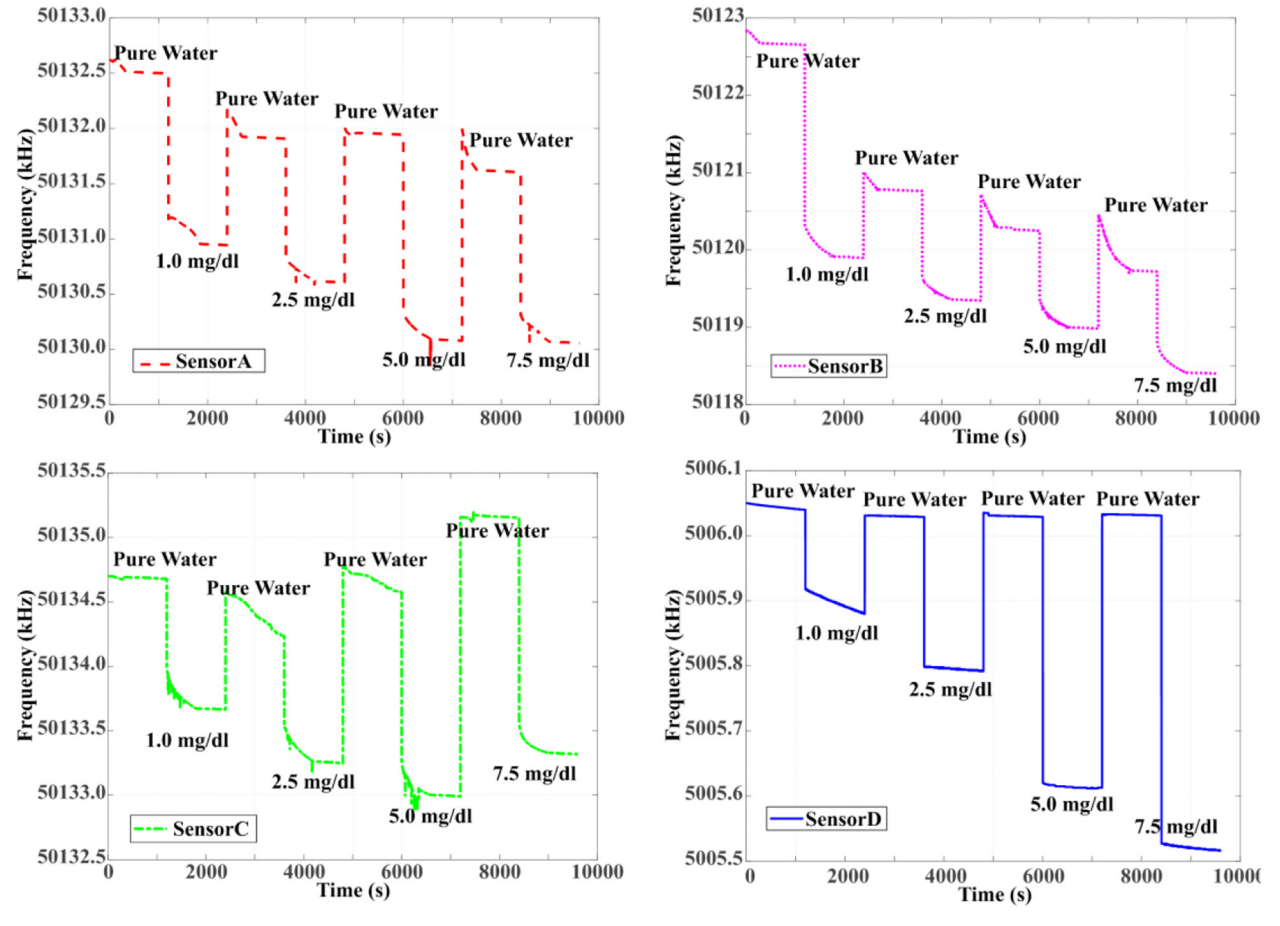
Four frequency responses of the different coated QCM sensors based on glucose concentration.

**Figure 8:**
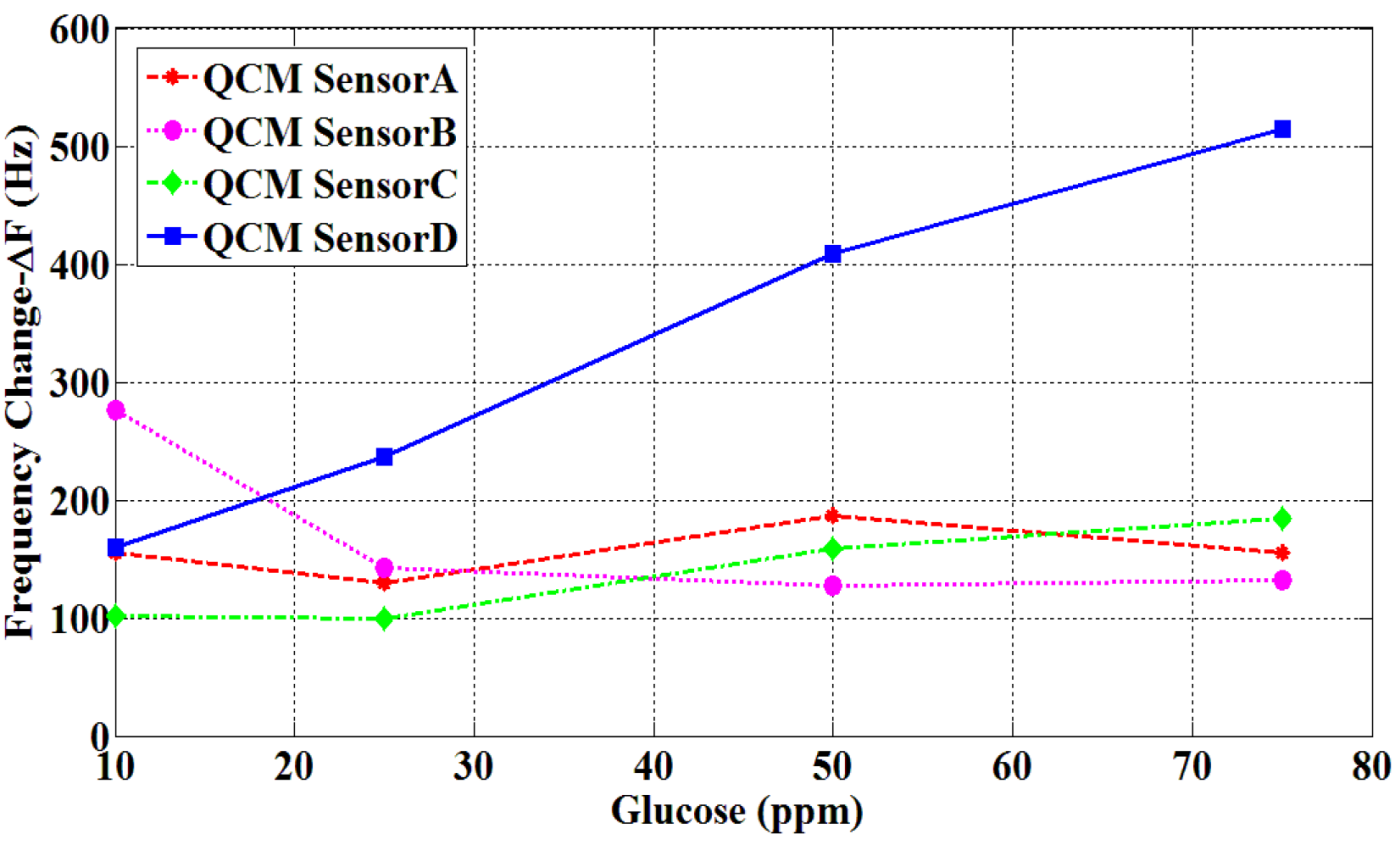
Comparative graphs of glucose/frequency values of sensors.

In order to identify the best performance for glucose, four different artificial glucose/pure water solutions of 1.0 mg/dL, 2.5 mg/dL, 5.0 mg/dL, and 7.5 mg/dL were put to four different coated QCM sensors. Frequency shifts for Sensor A, Sensor B, Sensor C, and Sensor D were observed as shown in Figure 7. Pure water and artificial glucose solutions were applied to the sensors for 20 min. Pure water was applied to sensors’ surfaces for cleaning purposes after each measurement. When QCM sensor results are examined, it can be seen that the responses to the four different concentrations of Sensor A, Sensor B, and Sensor C could not return to baseline frequencies. However, the responses of Sensor D returned to the baseline in each measurement. The Δf values for different concentrations of Sensor A, Sensor B, Sensor C, and Sensor D are shown in Figure 8. 

The response of Sensor D to glucose was obtained linearly across the entire concentration range. Accordingly, the response data were modeled using a linear fit function. Finally, it is observed that the best response to glucose is obtained with Sensor D. The sensitivity of sensor D was achieved as 5.46 Hz/ppm. The correlation coefficient for Sensor D is 0.995. According to these results, the most suitable sensor for determining glucose values of QCM sensor was defined as Sensor D in this study.

### 4.2. Human blood serum experiments

**Figure 9:**
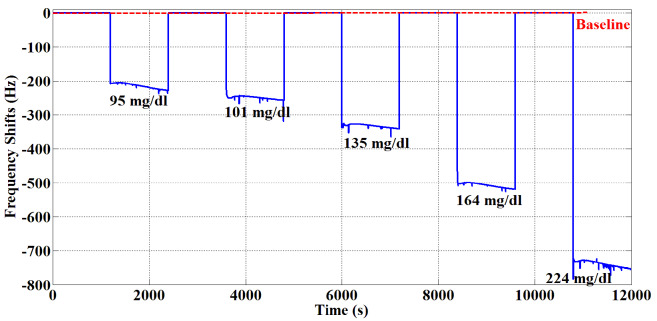
QCM sensor responses for five human blood serum samples.

At this stage, experiments were conducted to detect glucose in blood serum. As a result of experiments made with artificial glucose, Sensor D was found to display high performance. Then, the sensitivity and repeatability characteristic measurements of Sensor D were realized using blood serum. For sensitivity, five different blood glucose values (95 mg/dL, 101 mg/dL, 135 mg/dL, 164 mg/dL, and 224 mg/dL) and pure water were applied for 20 min to Sensor D during each measurement. Pure water was applied to Sensor D for cleaning purposes. As shown in Figure 9, Sensor D responded linearly to five different blood glucose values. As can be seen, the pure water frequency returned to baseline after each measurement.

**Figure 10:**
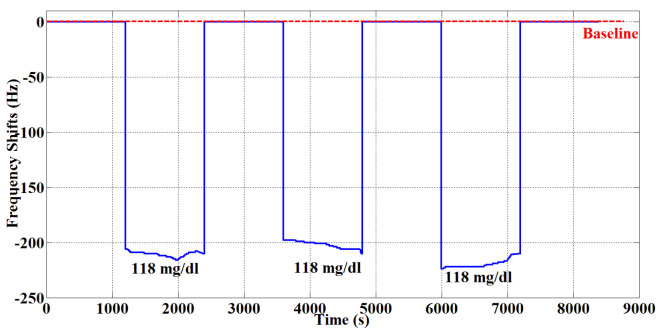
Repeatability of QCM sensor for blood serum.

To determine the repeatability of Sensor D, the same blood samples were evaluated three times consecutively as shown in Figure 10. Blood serum of 118 mg/dL was applied 3 times on Sensor D, and Sensor D was cleaned with pure water after each blood serum was applied. The sensor returned to baseline after each cleaning. According to Figures 9 and 10, it can be said that Sensor D is sensitive and repeatable for blood glucose.

**Figure 11:**
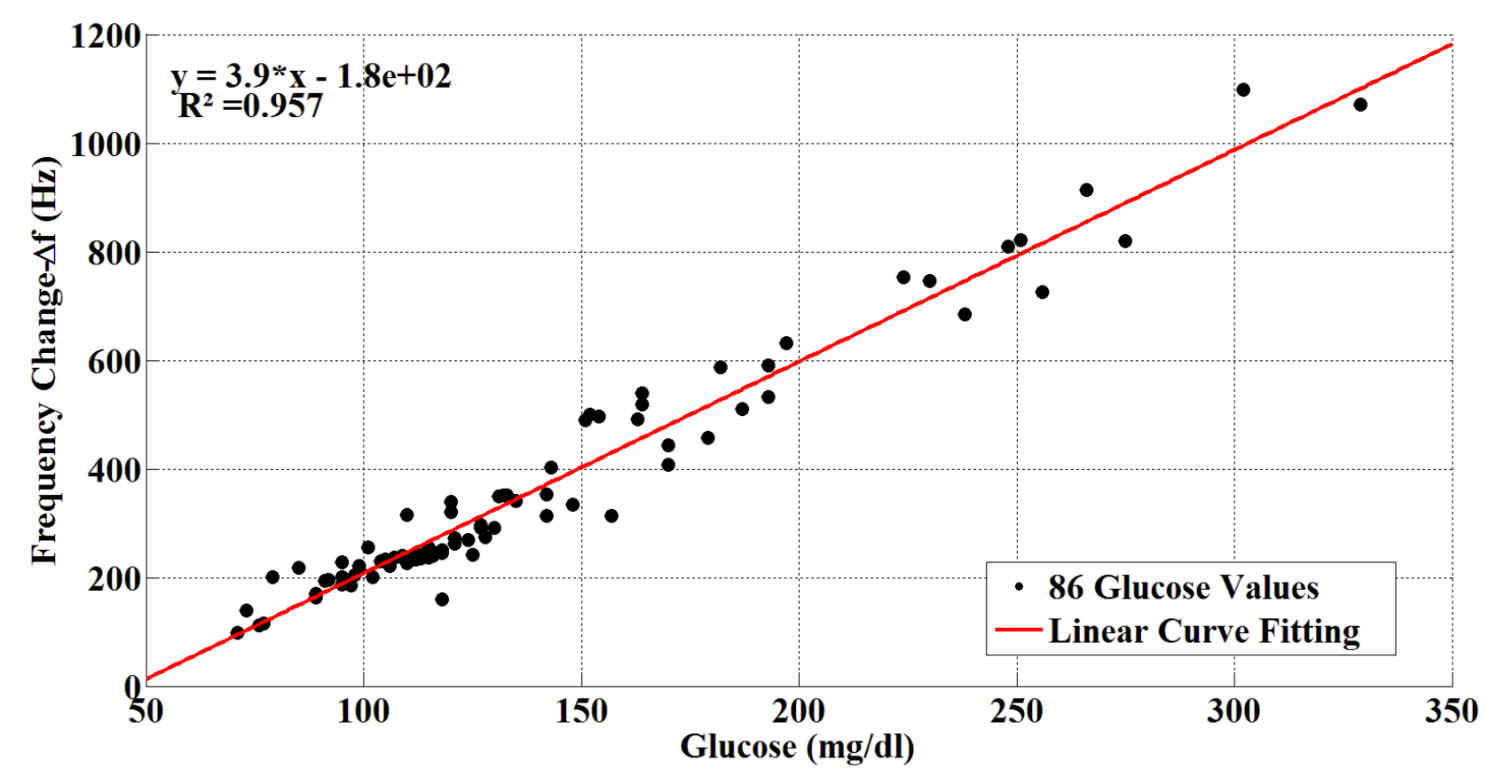
Glucose/frequency shifts chart for glucose values of 86 participants.

Human blood serum samples of 86 volunteers whose glucose levels ranged from 71 mg/dL to 329 mg/dL were used for the experiments. The average age of volunteers in the study was 56 (56 for men and 55 for women). The ∆f frequency values for volunteers are shown in Figure 11. As can be seen from Figure 11, the variation of sensor frequency shifts linearly through the different concentrations of blood glucose. The curved equation of the sensor response obtained by the curve fitting method is:

(2)y=3.9x-180

### 4.3. Sensitive materials

In this study, four different phthalocyanines (Pcs) were prepared and characterized for their artificial glucose sensing performance in water using the QCM sensors. After that, the phthalocyanine that gave the best result was chosen for blood glucose measurements. To achieve a broad diversity in the sorption properties, the Pc cores were chemically modified with substituents in peripheral position yielding tetra, octa, or hexadeca substituted Pcs and partly by introducing a central metal ion (Co^2+^, Cu^2+^, or TiO(IV)). The following substituents were attached to the Pcs core in peripheral position: Sensor A (TiOPc) HO-CH_2_-CF_2_-CHF_2_ [23], Sensor B (CuPc) HO-CH_2_-CF_3_ [24], Sensor C (CoPc) HO-CF_2_-CF_3_ [25],and Sensor D (CoPc) O-CH_2_-CHF_2_ that were previously described in detail [26].

## 5. Conclusion

In this research, a group of phthalocyanines was synthesized as sensitive materials for sensors operating directly in aqueous media and characterized for the detection and identification of glucose using QCM sensors. First, four different glucose/pure water concentrations were applied to four different sensors, and Sensor D was found to have the best performance. Then, Sensor D was used with human blood serums of 96 volunteers with blood glucose values ranging from 71 mg/dL to 329 mg/dL. Frequency shifts in the human blood serum of QCM sensors were observed. The linear sensor response was obtained based on the amount of glucose in the experiments. Data(∆F) obtained from 10 volunteers’ blood serum samples were reserved for the test and blood glucose values were determined using the interpolation method. For 10 blood serum samples, the glucose results of the biochemistry laboratory, and the glucose results of the proposed sensor assembly are given in Table comparatively and the relative error between them is calculated. The 10 biochemistry laboratory blood glucose values range from 90 mg/dL to 293 mg/dL. According to the Table, the minimum and maximum relative errors were0.32% and 9.48%. The average error rate was 3.25%. The correlation coefficient (r) of the sensors was 0.978, and the coefficient of determination was 0.957. 

**Table:** The comparison of the blood glucose levels of our measurements and biochemistry laboratory results.

Biochemistry lab. results (mg/dL)	Frequency change ∆F (Hz)	QCM glucose results (mg/dL)	Error rates (%)
90	165	89.00	1.11
95	215	99.00	4.21
105	235	113.00	7.62
119	253	116.50	2.10
130	36	132.78	2.14
154	497	153.50	0.32
174	455	177.20	1.84
196	591	193.00	1.53
219	689	239.76	9.48
293	965	286.73	2.13

As a result, the findings show a fast response and good linearity. In the present research, we found that the most active and stable phthalocyanine was Sensor D.

The glucose molecule has different physical and chemical properties, e.g. the molecules are capable of establishing different chemical interactions such as van-der-Waals forces (permanent dipole–dipole interactions and induced dipole interactions, London forces), π–π interactions, and hydrogen bonding. Hence, hydrogen atoms at the end of the substitute groups and central metal ions of phthalocyanine may cause some van-der-Waals interactions with the glucose molecule in the blood serum. However, to understand these interactions, we need some calculations and spectroscopic methods. Further studies should be done to investigate these interactions using RAMAN, FTIR, and DFT.
